# *QuickStats:* Percentage* of Women Aged 50–74 Years Who Had a Mammogram Within the Preceding 2 Years,^†^ by Family Income^§^ — National Health Interview Survey, United States, 2021^¶^

**DOI:** 10.15585/mmwr.mm7207a6

**Published:** 2023-02-17

**Figure:**
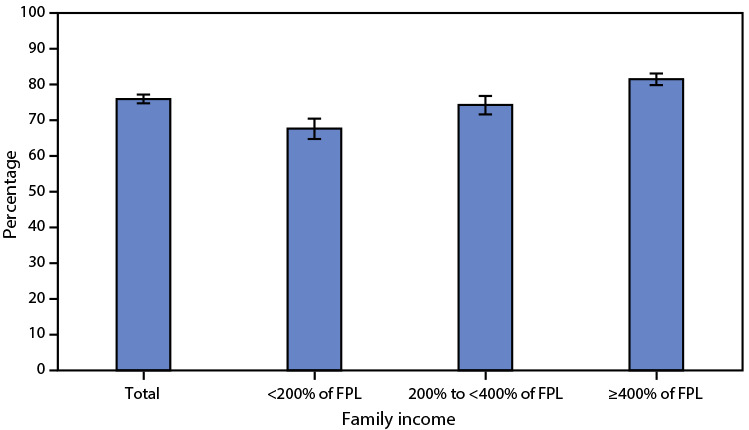
In 2021, 76.0% of women aged 50–74 years reported that they had a mammogram within the preceding 2 years. The percentage of women who had a mammogram within the preceding 2 years increased with family income, from 67.7% of women with family income <200% of FPL, to 74.3% of women with income 200% to <400% of FPL, and 81.5% of those with income ≥400% of FPL.

**Source:** National Center for Health Statistics, National Health Interview Survey, 2021. https://www.cdc.gov/nchs/nhis/index.htm

For more information on this topic, CDC recommends the following link: https://www.cdc.gov/cancer/dcpc/resources/features/breastcancerawareness/

